# Targeting the Unmet Need in Gastrointestinal Stromal Tumors: A Contemporary Review of Investigational Clinical Trials and Therapeutic Landscape

**DOI:** 10.3390/ph19040548

**Published:** 2026-03-29

**Authors:** Andrej Belančić, Juraj Prejac, Marin Golčić, Gordan Adžić, Andrija Katić, Lidija Kocić, Anamarija Kovač Peić, Nikša Librenjak, Borislav Belev, Ivana Mikolašević, Stjepko Pleština

**Affiliations:** 1 Department of Basic and Clinical Pharmacology and Toxicology, Faculty of Medicine, University of Rijeka, 51000 Rijeka, Croatia; 2 Department of Oncology, University Hospital Centre Zagreb, 10000 Zagreb, Croatia; juraj.prejac@gmail.com (J.P.);; 3 University of Zagreb, School of Dental Medicine, 10000 Zagreb, Croatia; 4 Tumor Clinic, Clinical Hospital Centre Rijeka, 51000 Rijeka, Croatia; 5 Department of Oncology and Radiotherapy, Faculty of Medicine, University of Rijeka, 51000 Rijeka, Croatia; 6 Department of Oncology and Radiotherapy, University Hospital Center Split, 21000 Split, Croatia; 7 School of Medicine, University of Split, 21000 Split, Croatia; 8 Department of Internal Medicine, General Hospital Pula, 52100 Pula, Croatia; 9 Department of Internal Medicine, General Hospital Slavonski Brod, 35000 Slavonski Brod, Croatia; 10 Department of internal medicine, Faculty of Medicine, University of Rijeka, 51000 Rijeka, Croatia; 11 Faculty of Medicine, University of Zagreb, 10000 Zagreb, Croatia

**Keywords:** gastrointestinal stromal tumour, tyrosine kinase inhibitors, clinical trials, targeted therapy

## Abstract

**Background**: Gastrointestinal stromal tumors (GISTs) are the most common mesenchymal neoplasms of the gastrointestinal tract and are primarily driven by activating mutations in KIT or PDGFRA. Although tyrosine kinase inhibitors (TKIs), particularly imatinib, have substantially improved outcomes, most patients with advanced disease eventually develop resistance, resulting in disease progression. **Methods**: We performed a narrative review with scoping approach of interventional clinical trials registered on ClinicalTrials.gov between January 2020 and July 2025 to characterize the contemporary investigational therapeutic landscape in GIST. Eligible studies included clinical trials evaluating novel agents, combinations, or alternative strategies beyond current regulatory approvals. Trial characteristics, therapeutic classes, endpoints, enrollment, and funding sources were analyzed. **Results**: A total of 27 ongoing trials were identified. Most studies were phase I/II and focused on metastatic or unresectable disease, predominantly in the second-line or later settings. TKIs remained the dominant therapeutic class, included in over 70% of trials, either as monotherapy or in combination. Emerging strategies comprised antibody–drug conjugates, immune checkpoint inhibitors, HIF inhibitors, FGFR inhibitors, and epigenetic modulators. Only four phase III trials were identified, reflecting the difficulty of conducting large, randomized studies in GIST. No trial used overall survival or quality of life as a primary endpoint. **Conclusions**: The current investigational landscape in GIST is largely focused on overcoming TKI resistance in advanced disease. Molecular stratification and personalized approaches dominate ongoing research, but evidence generation remains limited by small sample sizes and slow recruitment. Future trials integrating innovative therapeutic platforms and patient-centered outcomes are essential to improve long-term disease control and quality of life.

## 1. Introduction

Gastrointestinal stromal tumors (GISTs) are the most common mesenchymal neoplasms of the gastrointestinal tract. They share a common precursor or arise from interstitial cells of Cajal. Although GISTs are classified within the broader group of soft tissue sarcomas, they represent a distinct disease entity with unique biological drivers, clinical behavior, and therapeutic implications [1,2].

Most GISTs are characterized by activating mutations in the receptor tyrosine kinases CD117 (KIT) or platelet-derived growth factor receptor alpha (PDGFRA). GISTs predominantly affect adults and most commonly arise in the stomach, followed by the small intestine; other locations are less common [1,3]. The vast majority of cases occur sporadically, with hereditary syndromes accounting for only a small subset [4].

The clinical course of GIST is heterogeneous. While some tumors may be relatively indolent, others display aggressive behavior with early recurrence or metastatic spread. Prognosis depends on several factors, most importantly tumor size, mitotic activity, anatomical site, and tumor rupture, which form the basis of established risk-stratification systems. More recently, molecular genomic analysis has demonstrated both prognostic and predictive value in the management of GISTs [5]. Specific molecular variants confer distinct biological behavior; for instance, PDGFRA exon 18 D842V mutations are associated with indolent disease, whereas KIT exon 11 deletions involving codons 557–558 correlate with increased relapse risk [1,3,6,7].

Surgical resection remains the treatment of choice for localized disease [8]. Because patients with intermediate- and high-risk tumors face a substantial risk of recurrence, adjuvant imatinib, a tyrosine kinase inhibitor (TKI), administered for three years is currently established as the standard of care [9]. However, there is evidence in support of six years of adjuvant imatinib in patients with good tolerance [10]. The introduction of imatinib has also revolutionized the treatment of advanced GIST, enabling durable disease control in many patients and achieving a median overall survival well beyond five years. However, over time, most tumors acquire secondary resistance, necessitating treatment with other TKIs, such as sunitinib, regorafenib, and ripretinib, administered sequentially [1]. Specific cases, such as metastatic GIST lacking KIT/PDGFRA mutations or harboring PDGFRA D842V mutations, have different treatment algorithms, with avapritinib as the preferred option [1,11,12,13,14].

Despite the availability of multiple lines of therapy, long-term outcomes in advanced disease remain suboptimal, particularly in later lines of treatment, underscoring the need for more effective strategies to overcome resistance and achieve sustained disease control. Multidisciplinary care at high-volume centers is essential, as is access to clinical trials investigating novel agents and therapeutic strategies [1]. Hence, our study aims to characterize ongoing investigational therapies for GISTs by analyzing interventional trials registered on ClinicalTrials.gov. We sought to evaluate the therapeutic landscape beyond current standard treatments, with a particular focus on agents with potential regulatory or clinical impact.

## 2. Results

The majority of evaluated trials are actively recruiting (*N* = 22, 81.5%), with two (7.4%) not yet recruiting and three active but not recruiting (11.1%). While most trials are evaluating only GIST patients (*N* = 22, 81.5%), others are evaluating other tumors along with GIST. Furthermore, almost three-quarters of trials require a specific mutation profile (*N* = 20, 74.0%). Most trials evaluate patients in metastatic second-line treatment (*N* = 13, 48.1%), with only one trial evaluating a drug in the fifth line (*N* = 1, 3.7%) and four evaluating a first-line treatment (*N* = 4, 14.8%). The same number of trials evaluate patients in the neoadjuvant or adjuvant setting (Table 1).

**Figure 1:**
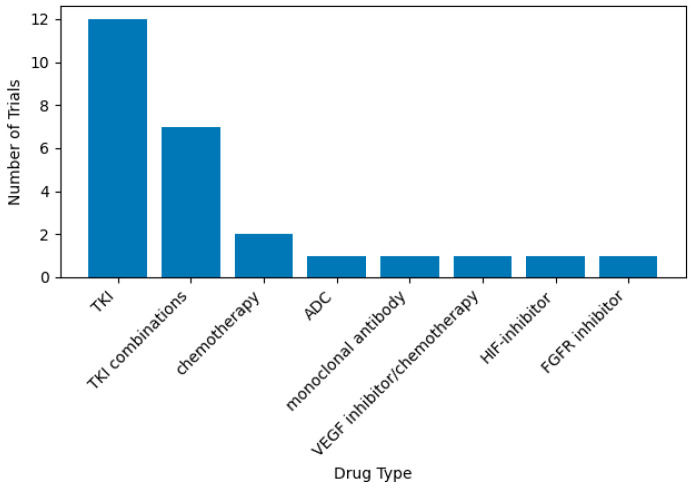
Distribution of different types of medications used in ongoing clinical trials for GIST patients.

TKIs remain the mainstay of research, with 12 trials (44.4%) evaluating TKI monotherapy, three more evaluating the TKI/TKI combination (11.1%), and four evaluating TKI with different drug types (14.9%), meaning that 19 (70.4%) of all trials include TKI. A single trial is evaluating ADC, monoclonal antibody, chemotherapy, HIF inhibitors, and FGFR inhibitor (all *N* = 1, 3.7%) (Figure 1).

Most of the trials are phase 2 (*N* = 16, 59.3%), with three more being phase 1/2 (11.1%). Only four trials are phase 3 (14.8%). Industry is responsible for 40.7% (*N* = 11) of trials, with other types of funders designated for the rest of the trials, and one is sponsored by the NIH (3.7%). The same number of trials are randomized and non-randomized, each representing 25.9% (*N* = 7), while for 13 (48.1%) of the trials the allocation is not specified. Over three-quarters (77.8%, *N* = 21) of the trials are conducted in a single country, while the rest are international. Similarly, most trials are multicenter (*N* = 17, 62.9%), with the remainder being single center (Table 1).

Planned enrollment ranges from 23 to 442 with a median of 67 patients (95% CI 47.5–110.0), while the estimated duration of trials ranges from 20 to 120 months (median 54 months, 95% CI 43.8–63.1). Industry trials enroll nearly twice as many participants as non-industry trials (median enrollment of 111 vs. 48, *p* = 0.0066). Additionally, there is a correlation between the number of countries involved in the trial and enrollment (R = 0.50, *p* = 0.0077) (Table 1).

**Figure 2:**
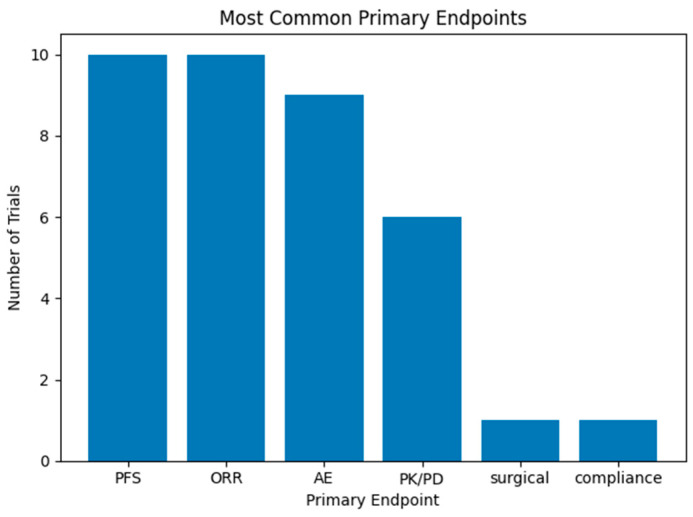
Most common primary endpoints in ongoing clinical trials for GIST patients. Trials can have more than one primary endpoint.

Primary endpoints included evaluating progression-free survival (PFS) (*N* = 10, 37.0%), response rate (RR) (*N* = 10, 37.0%), adverse effects (*N* = 9, 33.3%), pharmacokinetics, pharmacodynamics or drug dose (*N* = 6, 22.2%), and a single trial evaluated surgical outcomes (3.7%) or compliance (*N* = 1, 3.7%). No trials evaluated overall survival (OS) or quality of life (QoL) as a primary endpoint (Figure 2) (Table 1).

**Figure 3:**
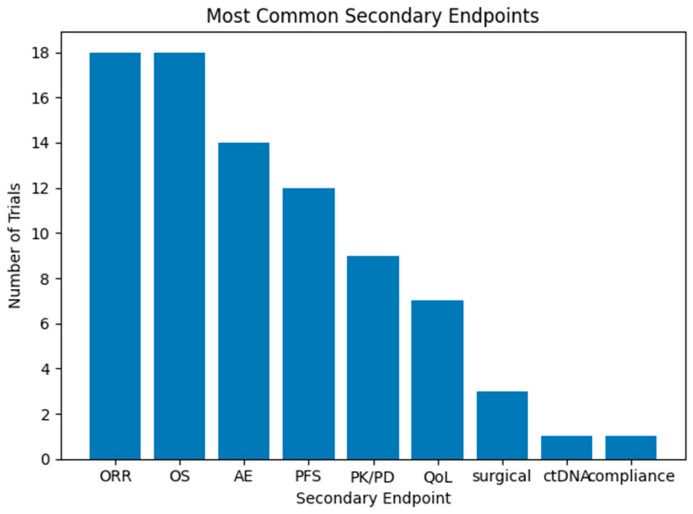
Most common secondary endpoints in ongoing clinical trials for GIST patients. Trials can have more than one secondary endpoint.

There were more reported secondary outcomes, which included RR (*N* = 18, 66.7%), OS (*N* = 18, 66.7%), adverse effects (*N* = 14, 51.8%), PFS (*N* = 12, 44.4%), pharmacokinetics, pharmacodynamics or drug dose (*N* = 9, 33.3%), QoL (*N* = 7, 25.9%), and surgical outcomes (*N* = 3, 11.1%). Additionally, a single trial evaluated ctDNA and compliance as a secondary outcome (each *N* = 1, 3.7%) (Figure 3) (Table 1).

## 3. Discussion

The review of ongoing clinical trials in GIST demonstrates the predominance of small early-phase clinical trials involving TKIs, predominantly in later therapy lines which reflects the biological, clinical, and logistical realities of GIST as a rare, molecularly driven disease.

The scarcity of phase III trials, with only four active phase III studies (14.8%), together with the marked difference in enrollment between industry- and non-industry-supported studies, underscores the difficulty of conducting large, randomized trials in this setting. Adequate patient accrual, therefore, appears to depend largely on industry support, as only one phase III trial in the dataset was conducted without industry sponsorship. The predominance of smaller early-phase trials and later-line metastatic populations is evident in the endpoint selection, with no studies using OS or QoL as a primary endpoint, while the most frequent primary outcomes were PFS and RR. This shows that rapid assessment of antitumor activity and tolerability is prioritized over evaluating long-term clinical benefit, which can be difficult to interpret because of prolonged post-progression survival and frequent crossover to subsequent therapies.

### 3.1. Approaches for GIST with Mutations in KIT/PDGFRa Pathways

**Figure 4:**
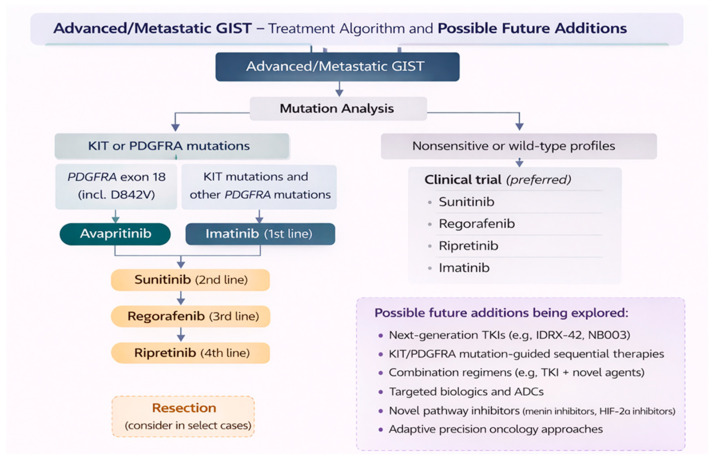
Treatment algorithm and possible future additions for the treatment of advanced or metastatic GIST.

Current international guidelines strongly recommend treating GIST based on molecular analysis and specific mutations [1] (Figure 4). Mechanistically, most investigational strategies continue to focus on the KIT/PDGFRA signaling axis, with 70.3% of trials including TKI or a TKI combination. While a smaller proportion of studies explore alternative approaches, including immune modulation, novel pathway inhibitions, and antibody–drug conjugates, the immediate future in GIST treatment, particularly in patients activating mutations in KIT (60–70%) and PDGFRA (10–15%) [15], will likely depend on the TKI. However, as resistance frequently arises through secondary, often polyclonal, mutations [16], there is an unmet need, especially in later therapy lines following first-line treatment with imatinib, which still represents the backbone first-line treatment of inoperable or metastatic GIST [1].

With several recent unsuccessful trials in the first line involving nilotinib [17] and regorafenib [18] and only four first-line trials (14.8%) ongoing, none of which are challenging imatinib in the first-line setting, the first-line choice in KIT-mutated GIST is likely not going to change soon. One of the first-line trials is exploring the use of imatinib beyond 10 years in the metastatic setting and the potential to discontinue imatinib treatment [19]. The previous phase III BFR14 trial showed that imatinib discontinuation in non-progressing patients with GIST was associated with rapid progression, faster resistance to imatinib, and shorter OS compared with imatinib continuation in patients after 3 years and 5 years of imatinib [20]. However, no data is available on discontinuation after 10 years, and the results of this ongoing trial could answer this burning question about the duration of treatment and even change treatment patterns.

An exception to the previously mentioned strategy is the presence of PDGFRA exon 18 D842V–mutant GIST, a subgroup historically resistant to imatinib and other TKIs, where the use of avapritinib demonstrated durable activity in the phase I NAVIGATOR trial [1,14].

For most patients with KIT/PDGFRa mutations, sunitinib is a standard of care upon progression to imatinib, based on a phase III trial that showed a benefit in PFS compared to placebo, with regorafenib and ripretinib reserved for subsequent lines [21,22,23] (Figure 4). Around half of current clinical trials in our review (48.1%) are evaluating therapeutic agents in the second-line setting, therefore reflecting the need for effective alternatives following imatinib failure beyond sunitinib.

Several phase II and phase III studies are evaluating combination strategies [24] such as bezuclastinib + sunitinib (Peak study (NCT05208047)) or next-generation TKIs such as IDRX-42 [25] in the post-imatinib setting. The Peak study has already published preliminary results, demonstrating that the combination regimen, compared to sunitinib alone, resulted in an ORR of 20% in the first 40 evaluated patients, with a manageable safety profile [26].

Of particular interest is INSIGHT, a phase III randomized study comparing ripretinib and sunitinib in imatinib-pretreated patients with advanced GIST carrying KIT exon 11 and exon 17 and/or 18 mutations [27]. It was designed following the INTRIGUE study, which compared ripretinib and sunitinib in an unselected post-imatinib population and failed to demonstrate superiority of ripretinib [28]. The INSIGHT trial explores whether this mechanism can translate into superior clinical benefit compared with sunitinib in a molecularly defined population enriched for secondary KIT exon 17 and 18 mutations, potentially supporting a mutation-guided approach to second-line therapy rather than a uniform sequence-based strategy.

However, there are some trials exploring completely different approaches to current standard treatments. Epigenetic modulation is one strategy being explored to overcome resistance in GIST [29]. A phase I study is evaluating ziftomenib, an oral menin inhibitor, in combination with imatinib, in patients with advanced GIST, representing an exploratory approach aimed at modulating transcriptional programs potentially involved in treatment resistance [30].

While limited clinical activity of PD-1/PD-L1 inhibitors was shown as monotherapy options in GISTs, novel approaches are evaluating the addition of atezolizumab to imatinib and regorafenib to envafolimab, with the idea of exposing tumor cells to TKIs while simultaneously enhancing T-cell activity and antitumor immune responses through PD-L1 blockade, thereby potentially sensitizing an otherwise immunologically “cold” GIST [31,32,33].

Pimitespib (TAS-116), a selective oral HSP90 inhibitor, has previously been investigated in GIST in a phase III CHAPTER-GIST-301 trial where it demonstrated a significant improvement in PFS compared with placebo, 2.8 vs. 1.4 months, respectively, and an acceptable safety profile in patients with advanced GIST refractory to at least three prior lines of TKI therapy [34]. It is now being evaluated in a phase I study with imatinib in patients with advanced GIST after progression on imatinib, with the aim of modulating resistance mechanisms [35].

Antibody–drug conjugates (ADCs) represent another emerging therapeutic strategy in GIST. NN3201, a c-KIT–specific ADC, is currently being evaluated in an early-phase study in patients with metastatic solid tumors known to express c-KIT, including GIST [36]. This therapeutic strategy targets c-KIT as a surface antigen rather than its kinase activity. The clinical value of ADCs in GIST, particularly in TKI-refractory disease, however, remains to be determined.

Finally, while conventional cytotoxic chemotherapy is not recommended in current treatment guidelines due to its limited clinical efficacy in the majority of GIST patients, several chemotherapy-based strategies are explored, mainly in heavily pretreated or molecularly profiled populations [37,38].

### 3.2. Approaches for Wild-Type GIST

The rise in trials evaluating specific mutation profiles in GIST (74.0% of trials) is welcome given the high unmet need. This is particularly true for GIST patients lacking common KIT or PDGFRA mutations (“wild-type” (wt) GIST) [17]. Wt GISTs are generally characterized by the lack of sensitivity to imatinib; therefore, they are in need of new alternative therapeutic strategies. The list of ongoing trials evaluating wt GIST is available in Table 2.

Succinate dehydrogenase (SDH)-deficient GISTs are the most common wt GISTs, usually developing during childhood or adolescence. Although sunitinib and regorafenib, through their antiangiogenic effects, lead to better responses than imatinib [39,40], treatment strategies remain suboptimal and finding more efficient medications is crucial. SDH loss causes succinate accumulation, which stabilizes hypoxia-inducible factors, creating a pseudohypoxic state by inducing angiogenic genes. Furthermore, it inhibits α-KG-dependent ten-eleven translocation (TET) enzymes, leading to DNA hypermethylation [41]. Hence, an approach targeting the HIF pathway could lead to clinical success. Belzutifan, a HIF-2α inhibitor, is primarily used to manage renal cell carcinoma (RCC) and Von Hippel–Lindau (VHL) disease-associated tumors, with phase III data supporting its use [42]. The molecular rationale to test this approach in GIST is based on the loss of SDH function [43], which leads to the pathological stabilization of HIF-2α, therefore driving tumor growth and proliferation [44].

The new third-generation TKIs olverembatinib and temozolomide, alkylating agents, also showed encouraging results in smaller studies [45,46]. Olverembatinib could be a particularly interesting development due to its effect on modulating lipid metabolism and thereby contributing to the drug’s antitumor effects [47], which represents a novel approach in this specific patient population. The current trial with olverembatinib (POLARIS-3) is recruiting patients with SDH-deficient GIST who have previously been treated [48].

Targeting the fibroblast growth factor receptor (FGFR) pathway has also emerged as an approach for SDH-deficient GIST. Rogaratinib is an orally bioavailable, highly potent, and selective FGFR1-4 kinase inhibitor that has been investigated in patients with various solid tumors harboring FGFR alterations [49]. A combination of rogaratinib and pemigatinib compared to sunitinib and regorafenib in SDH-deficient GIST demonstrated PR in ten out of 24 patients (41.7%) and stable disease in 12 patients (50%), with a median PFS of 31.1 months [50]. The type of SDH subunit did not affect the probability of an objective response, and adverse events showed the expected class effect typical of FGFR inhibitors. There is an ongoing study investigating rogaratinib in patients with sarcoma with FGFR alterations or SDH-deficient GIST [51].

An additional unmet need is the treatment of RAS pathway-mutated GIST, composed of neurofibromatosis 1-associated GIST, BRAF-mutated GIST, and extremely rare KRAS-mutated GIST [52]. These molecular subtypes are often associated with poor prognosis, resistance to imatinib and other TKIs, and difficulties with early trial closure due to slow accrual [53,54]. Furthermore, in a heterogeneous group of quadruple WT GIST (SDH-competent and without mutations in the RAS pathway), several genetic alterations (NTRK, ALK, FGF/FGFR/HER2) could be targeted in a tumor-agnostic approach, although trials remain scarce [52,55,56,57].

### 3.3. Barriers and Challenges

Most of what we know about the epidemiology of GIST comes from trials from a limited number of regions. Several key clinical trials included patients from Europe, North America, Australia, and Asia, with geographic region used as a stratification factor (EORTC 62005, SWOG S0033, CALGB 150105). Combined analyses from EORTC and SWOG represent one of the strongest datasets examining regional differences and showed that geographic region is not an independent prognostic factor, although a bias could still be present. Additionally, race is not consistently reported in GIST trials, and no study has shown that race independently affects response to tyrosine kinase inhibitors when mutation status is controlled for [40,58,59].

Slow patient recruitment is another persistent challenge in GIST clinical trials. This has been shown by long enrollment periods, small sample sizes, and the need for international collaboration in studies such as ACOSOG Z9001, EORTC 62005/SWOG S0033, and INVICTUS [29,40,58,59,60]. Recruitment difficulties affect trials across adjuvant, localized, and metastatic settings, particularly for rare subtypes, and are a major reason why evidence for the treatment of GIST is often based on small, pooled, global studies [61]. Our review confirms that the same issues persist, as current trials plan to enroll a median of 67 patients (95% CI 47.5–110.0), with a clear correlation between the number of countries involved in the trial and enrollment (R = 0.50, *p* = 0.0077). Furthermore, only six trials (22.2%) are being conducted internationally, with the rest being conducted in a single country.

One major reason is unequal access to proper diagnosis. A full diagnosis of GIST requires immunohistochemistry (KIT/CD117, DOG1) and molecular testing for KIT and PDGFRA mutations [1]. In many low-income regions, these tests are not routinely available, leading to misclassification of cases and hampering any inclusion in clinical trials, which in almost 75% of cases require a specific mutational profile. One study reported that 20% of initially reported quadruple Wt GISTs harbored a KIT mutation detected by deep sequencing, demonstrating the necessity of incorporating deep sequencing in molecularly driven trials [62]. Hence, it is not surprising that most clinical trials are conducted in high-income countries and that many studies rely on data from tertiary referral centers rather than population-based samples [52,60,63,64].

Access to targeted therapies also varies widely, and many countries lack cancer registries or reliable health statistics. In the past, the epidemiological evaluation of GIST was further limited by the absence of clear diagnostic criteria and a specific International Classification of Diseases code, meaning that only clinically clear malignant cases were recorded. Current estimates suggest that prevalence is more than ten times higher than incidence, with approximately 135–155 GIST survivors per million people reported in several studies [65].

Considering the above, patient associations and international consortia play a key role in rare tumor research by helping to identify and enroll patients, raise awareness, and raise funds, shaping patient-centered outcomes. This facilitates shortening recruitment time and expanding access to studies [66]. Indeed, disease-specific platforms and adaptive designs prove to be efficient but still underutilized models for accelerating drug development and enable the simultaneous testing of multiple candidates in small, molecularly heterogeneous populations such as GIST [67]. Examples and methodological analyses show that such approaches reduce the time to conclusions and improve research efficiency.

Additional comments are required regarding the use of endpoints in the trials analyzed. Most trials are reliant on RR or PFS as primary endpoints. Both are widely accepted surrogate endpoints in advanced GIST, and regulatory agencies such as the Food and Drug Administration (FDA) and the European Medicines Agency (EMA) have historically accepted them for drug approval in refractory GIST based on unmet need, feasibility constraints, and the rarity of the disease [68,69]. However, their ability to reliably predict OS and QoL remains uncertain. This limitation is especially relevant in GIST, where prolonged post-progression survival and sequential TKI use may dilute any observable OS benefit attributable to a single agent, and formal validation of these surrogates as predictors of long-term patient-centered benefit remains limited [70,71].

### 3.4. Limitations

Although our study relies on ClinicalTrials.gov, which is the most comprehensive global database, we recognize that some trials registered exclusively in regional registries (e.g., EUCTR and ChiCTR) may not have been captured; however, based on previous experience and Supplementary Data showing overlapping registry identifiers, the risk of missing relevant studies is likely minimal.

### 3.5. Future Directions and Conclusions

Most ongoing trials evaluate second and later lines of metastatic or unresectable GIST, often targeting specific molecular subtypes and resistance mechanisms. The personalization of treatment strategy is key and should be based on the determination of gene alterations by next-generation sequencing and repeated biopsies in order to account for the evaluation of secondary mutations [62]. This process, however, is not only time-consuming and expensive but also sometimes not possible and susceptible to many factors.

Innovations in artificial intelligence (AI) and liquid biopsy will probably be valuable tools in future GIST treatment, with AI being used and integrated to interpret radiological and pathohistological images and data from molecular analyzes, and to integrate inputs from individual stakeholders involved in patients’ diagnosis and treatment. This technology saves time and enables faster decision-making and optimal patient treatment [71,72]. Kong et al. developed an AI-based deep learning model, a potential model for predicting responses to TKI, that uses convolutional neural networks to analyze digitized hematoxylin and eosin staining in tumor histological sections and predicts responses to imatinib or avapritinib treatment [71].

AI models that use real clinical and pathological data to recommend the duration of adjuvant imatinib treatment after GIST resection reduce risk and avoid unnecessary toxicity by optimizing the balance of benefits and harms for individual patients [73]. Indeed, liquid biopsy and detection of circulating tumor DNA (ctDNA) are emerging as noninvasive biomarkers in the management of different tumor types. Its uses include molecular profiling, monitoring treatment response, detecting resistance mutations, and assessing minimal residual disease and risk of recurrence. In GIST, ctDNA analysis enables the detection of primary driver mutations, mainly KIT and PDGFRA, as well as secondary resistance mutations [74,75,76]. Serial ctDNA measurements can be used to track disease activity, detecting progression often preceding radiological confirmation, and can identify clonal evolution, simultaneously compensating for intratumoral heterogeneity [77,78]. To enhance ctDNA analysis, implementing AI technology would improve mutation detection sensitivity and the integration of multi-omics data for prognostic and predictive modeling [72,79].

The optimal use of ctDNA in the future as a surrogate marker assumes standardization of analytical methods, validated panels, low detection sensitivity, and clearly set thresholds. Clinical decision-making will be based on testing such an approach in prospective and adaptive clinical studies [80].

In parallel with the above, it is necessary to develop, standardize, and prospectively validate AI models that integrate all relevant data, stratify patients, assess disease progression risk, and predict response to therapy [81,82].

That being said, the clinical application of AI technology in practice faces several obstacles, primarily due to the regulatory framework and the lack of standardized procedures and interoperability across different systems. Furthermore, the very complexity of this technology and the dependence on individual inputs make it difficult for doctors to interpret the findings [72,83].

Furthermore, while novel drugs in development often target a spectrum of secondary mutations and multiple pathways, combining systemic therapy with surgical approaches [84,85], locally ablative methods [86], and radiotherapy [87] could potentially improve the effectiveness in treating metastatic GIST in the near future.

However, larger trials on this topic could be hampered by difficulties in patient enrollment and potential lack of interest from industry. Nonetheless, personalized, tailored treatments with multidisciplinary care based on molecular findings and resistance mechanisms remain the crucial approach to GIST patients, and further research on this topic should lead to new treatment strategies and, importantly, an improvement in patient-related and oncological outcomes. Importantly, with the abundance of new medications, a placebo should not be used as a comparator in the majority of further trials. Last but not least, studies prioritizing QoL and patient-reported outcomes are essential to complement clinical advances and should be incorporated into future GIST trials, following standards like those from the SISAQOL-IMI initiative to ensure holistic patient care [88].

## 4. Materials and Methods

We conducted a comprehensive search of ClinicalTrials.gov [89], the largest international registry of randomized controlled trials (RCTs), to identify investigational therapies for GIST. ClinicalTrials.gov lists over 300,000 studies globally, compared with approximately one-tenth that number registered in the European Union Clinical Trials Register [90]. Given the global nature of multicenter RCTs evaluating novel therapeutics—particularly those seeking regulatory approval in the United States, Europe, and other jurisdictions—ClinicalTrials.gov was deemed the most appropriate primary data source for this analysis.

On 28 July 2025, two independent reviewers (A.B. and M.G.) searched ClinicalTrials.gov using the following parameters: (i) indication: GIST, including the terms “GIST” and “Gastrointestinal Stromal Tumor”; (ii) recruitment status: not yet recruiting, recruiting, or active but not recruiting; (iii) population age: adult (18–64 years) and older adult (≥65 years); (iv) study phase: early phase 1, phase 1, phase 2, or phase 3; (v) study type: interventional; and (vi) study start date: on or after 1 January 2020. Eligible trials were restricted to those investigating novel therapeutic strategies. These included agents not yet approved by the US Food and Drug Administration (FDA) or the European Medicines Agency (EMA) for the treatment of GIST, alternative dosing regimens (e.g., doses exceeding current approvals), and studies exploring novel routes of administration or drug delivery systems. Discrepancies between the two independent reviewers were minimal (<5%) and were resolved through discussion. In cases where consensus could not be reached, a third author (B.B.) made the final decision.

A five-year capture window was selected to allow sufficient time to identify and develop candidate therapies that may progress to regulatory review. This interval reflects typical timelines for interim analyses, developmental decision-making in active trials, and regulatory milestones [91]. All data were extracted into a pre-specified Microsoft Excel (Redmond, WA, USA) spreadsheet designed for this study. Extracted fields included: NCT number and other identifiers, study title, study URL, phase, geographic location, investigational substance or intervention, eligibility criteria, study outcomes, study start and completion dates, anticipated or actual enrollment, recruitment status, sponsor, and collaborators (Supplementary Table S1).

## Figures and Tables

**Table 1:** List of ongoing clinical trials with details involving patients with GIST.

NCT Number	Study Status	Tumors Evaluated	Specific Mutation Profile	Earliest Line of Treatment	Investigated Drug Name	Investigated Drug Type	Sponsor	Phase	Enrollment (Estimated)	Funder Type	Allocation	Number of Countries	Single/ Multicentered	Planned Duration (Months)
NCT06805825	RE	GIST and other	no	2	NN3201	ADC	Novelty Nobility, Inc.	1	67	industry	NR	1	MC	34
NCT06772233	RE	GIST	yes	5	regorafenib with envafolimab vs. physician choice	PD-L1/TKI	Peking University	2	82	other	R	1	MC	36
NCT06760819	RE	GIST and other	yes	2	BAY2927088	TKI	Bayer	2	111	industry	NR	11	MC	32
NCT06655246	RE	GIST	yes	2	ziftomenib with imatinib	Oral menin inhibitor/TKI	Kura Oncology, Inc.	1	157	industry	NR	1	MC	44
NCT06640361	RE	GIST	yes	2	olverembatinib	TKI	Ascentage Pharma Group Inc.	3	40	industry	n/a	1	SC	55
NCT06630234	RE	GIST	no	2	DCC-3009	TKI	Deciphera Pharmaceuticals, LLC	1/2	120	industry	n/a	1	MC	40
NCT06380816	RE	GIST and other	yes	n.s.	UCB4594	monoclonal antibody	Cancer Research UK	1/2	167	other	NR	1	MC	63
NCT06326346	not yet RE	GIST	yes	4	liporaxel	chemotherapy	Asan Medical Center	2	28	other	n/a	1	SC	31
NCT06208748	active, not RE	GIST	yes	3	bezuclastinib with sunitinib	TKI/TKI	Sarcoma Alliance for Research through Collaboration	2	40	other	n/a	1	MC	34
NCT06087263	RE	GIST	yes	2	regorafenib	TKI	M.D. Anderson Cancer Center	2	30	other	n/a	1	SC	120
NCT05970900	not yet RE	GIST	no	n/a	imatinib	TKI	Fujian Medical University Union Hospital	3	23	other	n/a	1	SC	72
NCT05957367	RE	GIST	no	2	inlexisertib with ripretinib	TKI/TKI	Deciphera Pharmaceuticals, LLC	1/2	94	industry	NR	1	MC	65
NCT05905887	RE	GIST	yes	4	Rivoceranib with paklitaxel	VEGF inhibitor/chemotherapy	Asan Medical Center	2	48	other	n/a	1	SC	39
NCT05734105	RE	GIST	yes	2	ripretinib vs. sunitinib	TKI	Deciphera Pharmaceuticals, LLC	3	54	industry	R	15	MC	47
NCT05661643	RE	GIST	yes	2	temozolomide	chemotherapy	Asan Medical Center	2	29	other	n/a	1	SC	54
NCT05500391	RE	GIST and other	no	A	follow-up only	follow up	Centre Oscar Lambret	2	88	other	R	1	MC	67
NCT05493215	RE	GIST	yes	1	imatinib (monitoring only)	TKI	Reema A. Patel	2	28	other	n/a	1	SC	20
NCT05489237	RE	GIST	yes	2	IDRX-42	TKI	IDRx, Inc.	1	269	industry	NR	11	MC	49
NCT05385549	RE	GIST	no	A	imatinib	TKI	Asan Medical Center	2	35	other	n/a	1	SC	91
NCT05366816	RE	GIST	yes	2	sunitinib or regorafenib	TKI	University of Miami	2	48	other	NR	1	SC	62
NCT05245968	RE	GIST	no	2	pimitespib with imatinib vs. sunitinib (reference data)	HSP90 inhibitor/TKI	Taiho Pharmaceutical Co., Ltd.	1	78	industry	R	5	MC	48
NCT05208047	active, not RE	GIST	yes	2	Bezuclastinib + sunitinib (one arm with midazolam) vs. sunitinib	TKI/TKI	Cogent Biosciences, Inc.	3	442	industry	R	22	MC	52
NCT05152472	RE	GIST	yes	4	Imatinib + atezolizumab vs. imatinib	PD-L1/TKI	Centre Leon Berard	2	110	other	R	1	MC	68
NCT05009927	RE	GIST	yes	1	imatinib (discontinue/continue after 10 years)	TKI	Centre Leon Berard	2	50	other	R	1	MC	59
NCT04933669	RE	GIST	yes	NA	imatinib	TKI	First Affiliated Hospital of Zhejiang University	2	122	other	n/a	1	SC	99
NCT04924075	RE	GIST and other	yes	1	belzutifan	HIF-inhibitor	Merck Sharp & Dohme LLC	2	322	industry	n/a	22	MC	93
NCT04595747	active, not RE	GIST	yes	1	rogaratinib	FGFR inhibitor	National Cancer Institute (NCI)	2	48	nih	n/a	1	MC	61

ADC = antibody–drug conjugate, FGFR = fibroblast growth factor receptor, GIST = gastrointestinal stromal tumors, HIF = hypoxia induced factor, HSP = heat-shock protein, MC = multicentered, PD-L1 = programmed death ligand 1, RE = recruiting, R = randomized, NR = non-randomized, TKI = thyrosine kinase inhibitor, SC = single-centered, VEGF = vascular endothelial growth factor, n/a = not available or not specified.

**Table 2:** A summary of selected trials targeting wild-type GIST.

NCT Number	Molecular Subtype	Investigated Drug	Drug Class	Phase	Earliest Line Allowed	Primary Endpoint	Sponsor
NCT04924075	Wild-type GIST (SDH-deficient included)	Belzutifan	HIF-2α inhibitor	II	1	Objective response rate	Merck Sharp & Dohme
NCT04595747	SDH-deficient GIST	Rogaratinib	FGFR1-4 inhibitor	II	≥1	Objective radiographic response rate	National Cancer Institute
NCT06640361	SDH-deficient GIST	Olverembatinib	3rd-generation TKI	III	≥2	Progression-free survival	Ascentage Pharma Group Inc.
NCT05661643	SDH-deficient GIST	Temozolomide	Alkylating agent	II	≥2	Objective response rate	Asan Medical Center
NCT06772233	KIT exon 17 mutant GIST	Regorafenib + Envafolimab	TKI + PD-L1 inhibitor	II	≥5	Progression-free survival	Peking University
NCT05734105	KIT exon 11 + exon 17/18 mutations	Ripretinib vs. Sunitinib	Switch-control TKI	III	2	Progression-free survival	Deciphera Pharmaceuticals
NCT06087263	KIT exon 14, 17, 18 mutations or SDHB-deficient GIST	Regorafenib	TKI	II	2	Progression-free survival	MD Anderson Cancer Center

## Data Availability

No new data were created or analyzed in this study. Data sharing is not applicable to this article.

## Supplementary Materials

The supplementary file has been added and can be downloaded at: https://www.mdpi.com/article/10.3390/ph19040548/s1, Table S1: Clinical Trial Information Table.
